# Monotherapy With Anti-CD70 Antibody Causes Long-Term Mouse Cardiac Allograft Acceptance With Induction of Tolerogenic Dendritic Cells

**DOI:** 10.3389/fimmu.2020.555996

**Published:** 2021-02-19

**Authors:** Jing Zhao, Weitao Que, Xiaoxiao Du, Masayuki Fujino, Naotsugu Ichimaru, Hisashi Ueta, Nobuko Tokuda, Wen-zhi Guo, Piotr Zabrocki, Hans de Haard, Norio Nonomura, Xiao-Kang Li

**Affiliations:** ^1^ Division of Transplantation Immunology, National Research Institute for Child Health and Development, Tokyo, Japan; ^2^ Department of Specific Organ Regulation (Urology), Osaka University Graduate School of Medicine, Osaka, Japan; ^3^ Department of General Surgery, The First Affiliated Hospital of Soochow University, Suzhou, China; ^4^ Department of Hepatobiliary and Pancreatic Surgery, The First Affiliated Hospital of Zhengzhou University, Zhengzhou, China; ^5^ AIDS Research Center, National Institute of Infectious Diseases, Tokyo, Japan; ^6^ Department of Anatomy (Macro), Dokkyo Medical University, Tochigi, Japan; ^7^ argenx BV, Zwijnaarde, Belgium

**Keywords:** allograft, CD70, dendritic cell, regulatory T cell, rejection, tolerance

## Abstract

Allograft rejection has been an obstacle for the long-term survival of patients. CD70, a tumor necrosis factor (TNF) family member critically expressed on antigen-presenting cells and strongly but transiently up-regulated during lymphocyte activation, represents an important co-stimulatory molecule that induces effective T cell responses. We used a mouse heterotopic cardiac transplantation model to evaluate the effects of monotherapy with the antibody targeting mouse CD70 (FR70) on transplantation tolerance and its immunoregulatory activity. FR70-treated C3H recipient mice permanently accepted B6 fully mismatched cardiac allografts. Consistent with the graft survival, the infiltration of CD8^+^ T cells in the graft was reduced, dendritic cells were differentiated into a tolerogenic status, and the number of regulatory T cells was elevated both in the graft and the recipient’s spleen. In addition, naïve C3H given an adoptive transfer of spleen cells from the primary recipients with FR70 treatment accepted a heart graft from a matching B6 donor but not third-party BALB/c mice.　Our findings show that treatment with FR70 induced regulatory cells and inhibited cytotoxic T cell proliferation, which led to long-term acceptance of mouse cardiac allografts. These findings highlight the potential role of anti-CD70 antibodies as a clinically effective treatment for allograft rejection.

## Introduction

Significant breakthroughs have been made in regard to the short-term graft survival over the last few decades, but improving the long-term graft survival still remains a challenge. Innovative treatments preventing transplant rejection, in particular, is a significant unmet need (1). Previous studies have shown that CD70 blockade can inhibit the clonal expansion of CD8^+^ T cells and reduce the generation of memory CD8^+^ T cells (2, 3). Anti-CD70 monoclonal antibody (mAb) administered in combination with an anti-CD154/LFA-1 mAbs regimen during primary transplantation alleviated the accelerated rejection mediated by memory T cells (4). Furthermore, in a memory T cell-based adoptive mouse model, the combination of four antibodies against CD44, CD70, CD40L, and LFA-1 significantly inhibited the proliferation of memory T cells *ex vivo* but failed to induce long-lived heart allograft acceptance *in vivo* (5).

Recently, the role of suppressive signaling from dendritic cells (DCs) inducing graft tolerance has been emphasized (6, 7). The CD70 levels in mice are transiently up-regulated during lymphocyte activation, making it difficult to track its expression on DCs (8, 9). Nevertheless, CD70 expressed on DCs during their maturation plays a critical role in immunogenicity while also playing a pivotal role in priming T cells and in the function of regulatory T cells (10). Indeed, the transgenic expression of CD70 on resting DCs is sufficient to provide CD8^+^ T cells with immunity, whereas its blockade averts CD8^+^ T cell priming in lymphocytic choriomeningitis virus infection (8, 9, 11, 12). In addition, anti-CD70 treatment was able to suppress Th17-cell induced inflammatory diseases, including experimental autoimmune encephalomyelitis (13). In cancer vaccine therapy, blocking CD70 markedly reduced the effect of CD4^+^ T cell-mediated aid, such that CD70 blockade almost completely abolished the therapeutic effect of the “Help” vaccine (14).

DCs are the most potent antigen-presenting cells and play a key role in the immune response, both in tolerance and immunity (15, 16). Recent studies have shown that regulatory DCs primarily induce tolerance *via* the generation of regulatory T cells (Tregs) and the inhibition of T cells with their immature status *in vivo* (17, 18). In our previous study, regulatory DCs generated from induced pluripotent stem cells (iPSCs) were useful as an immune suppressive vaccine for inducing the generation of alloantigen-specific Tregs, and ensuring the long-term acceptance of mouse cardiac allografts (19, 20).

In the present study, we explored the CD70-mediated mechanism underlying the long-term graft survival. The treatment of allogenic graft recipients with an antibody targeting mouse CD70 (FR70) induced tolerogenic DCs (TolDCs) and Tregs, resulting in a decreased cytotoxic T lymphocyte (CTL) proliferation despite the presence of alloantigens, and thus inducing the long-term acceptance of a cardiac allograft in mice.

## Materials and Methods

### Animals and Mouse Heterotopic Cardiac Transplantation Model

All animals in this experiment were used in accordance with the recommendations in the Guide for the Care and Use of Laboratory Animals of the National Institutes of Health. Male C57BL/6NCrSlc (B6, H-2^b^) mice, C3H/HeNSlc (C3H, H-2^k^) mice, BALB/cCrSlc (BALB/c, H-2^d^) mice, 8–12 weeks old, were purchased from SLC (Shizuoka, Japan). All of the mice were kept under specific-pathogen-free conditions. The heart transplantation procedures were conducted as previously described (21). The experimental protocol was approved by the Committee on the Ethics of Animal Experiments of the National Research Institute for Child Health and Development, Tokyo, Japan.

### Reagents

Anti-mouse CD70 antibody (mouse IgG2a, FR70) and the isotype control were supplied by argenx BV (Zwijnaarde, Belgium).

### Treatment Protocol

Mice received heterotopic cardiac transplantation and were separated into three groups: the syngeneic group, control group, and treatment group. In the treatment group, mice were treated with intraperitoneal (i.p.) injection of 500 µg FR70 immediately after the surgery and with three half-dose (250 µg/mouse, i.p.) injections in the following 5 days. The control group was treated with an isotype control IgG. The cardiac graft survival was determined by the daily palpation of the recipient’s abdomen. An impalpable heart beat was considered graft rejection and confirmed visually by laparotomy.

### Adoptive Transfer Studies

Spleen cells (SPCs, 5 × 10^7^) from primary recipient C3H mice bearing an accepted B6 cardiac allograft on post-operative day (POD) 60 and 100 were adoptively transferred to secondary recipient C3H mice. The secondary recipient mice underwent transplantations with B6 (donor-type) or BALB/c (third-party) cardiac grafts immediately after the splenocytes had been injected.

### Isolation of Graft-Infiltrating Lymphocytes and Flow Cytometry Analysis

Graft-infiltrating lymphocytes (GILs) were isolated from the cardiac graft, and a flow cytometry (FCM) analysis was performed, as previously described (22–24). Spleen tissue and GILs were stained with fluorochrome-conjugated anti-CD3 (145-2C11), anti-CD4 (GK1.5), anti-CD8 (53-6.7), anti-CD25 (PC61), anti-CD44 (IM7) and anti-CD62L (MEL-14) mAb; for the DC analysis, cells were stained with anti-CD11b (M1-70), anti-CD11c (N418), anti-CD40 (3/23), anti-CD80 (16-10A1), anti-CD86 (GL-1), anti-IA/IE (M5/114.15.2), and anti-PD-L1 (10F.9G2) conjugated with a particular fluorochrome, and corresponding isotype controls were used (BioLegend, Inc., San Diego, CA, USA). Intracellular Foxp3 staining was performed using a BD Cytofix/Cytoperm Kit and anti-Foxp3 mAb (FJK-16s) (eBioscience, San Jose, CA, USA). The analysis of stained cells was performed with an LSRFortessa system (BD Biosciences), and the data were analyzed using the FlowJo software (Version 10.5.0; BD Biosciences). Detailed gating strategies are presented in **Supplementary Figures 2** and **3**.

### RNA Isolation and Quantitative Real Time Reverse Transcription Polymerase Chain Reaction

In order to measure the target expression, total RNA was isolated from SPCs and GILs using ISOGEN (Nippon Gene, Tokyo, Japan) according to the manufacturer’s protocol, with the detailed procedure described elsewhere (23, 25, 26).

### Histological Analysis

Grafts were dissected and fixed in 4% paraformaldehyde for 2 days, embedded in paraffin, and cut into 4-μm-thick sections. The sections were then stained with hematoxylin and eosin (H&E).

### Immunohistochemistry

Grafts were harvested on POD7 and embedded in Tissue-Tek^®^ O.C.T Compound. To label proliferating cells, recipient mice received intravenous injection of 5-bromo-2′-deoxyuridine (BrdU, Sigma-Aldrich, St Louis, MO; 0.6 mg/mouse) in sterile PBS 1 h before sampling. Triple immunoenzyme staining of 4-µm-thick cryosection was performed as previously described (27) with minor modifications. In brief, rat anti-mouse CD8*α* mAb (clone 53–6.7, dilution 1:100; BioLegend) was used as the primary antibody. As the secondary antibody, ALP-conjugated donkey anti-rat Ig (Jackson Immuno Research, West Grove, PA) was used at a 1:100 dilution in PBS-0.2%BSA supplemented with 1% heat-inactivated normal mouse serum (Thermo Fischer Scientific Inc, Waltham, MA). After visualizing CD8*α*-positive cells in blue using a Vector-Blue kit (VECTOR Laboratories, Burlingame, UK), the sections were further stained in brown for type IV collagen and finally in red for BrdU, as previously described (27). Images were captured by a DP26 camera using the CellSens software program (Olympus, Tokyo, Japan).

### Statistical Analysis

All data were analyzed by GraphPad Prism 8 (GraphPad Prism Software Inc., San Diego, CA, USA) and presented as the mean ± SEM. Student’s *t*-test, the Mann–Whitney test, or a one-way analysis of variance was performed to determine significant differences among groups. The graft survival was statistically analyzed by a Kaplan–Meier curve, and the log rank test was performed to determine the effect. Significance was reported at *p<*0.05.

## Results

### Treatment With FR70 Induces Long-Term Acceptance of Mouse Cardiac Allografts

**Figure 1:**
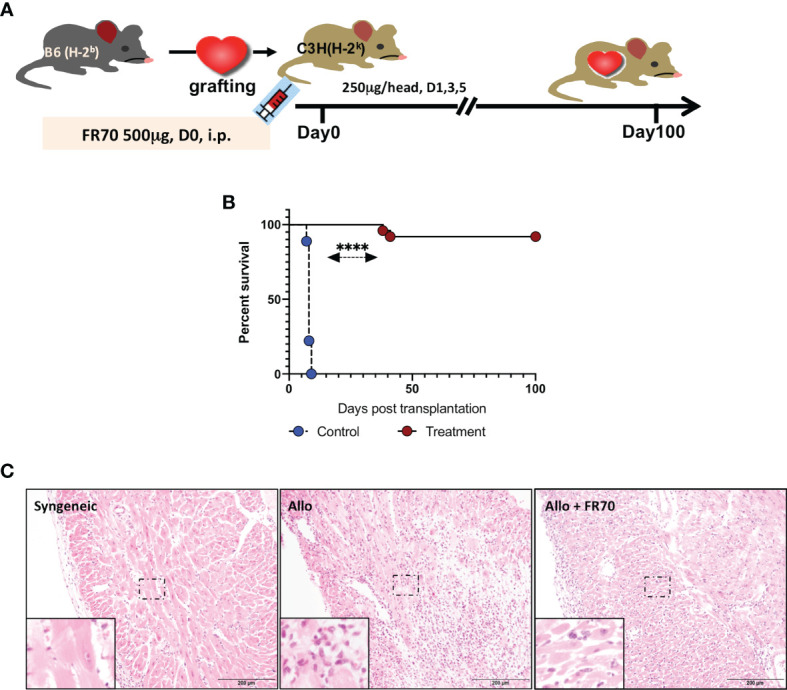
Anti-CD70 antibody (FR70) treatment induced long-term acceptance of mouse cardiac grafts. **(A)** FR70 (500 µg) was intraperitoneally (i.p.) injected into C3H recipient mice immediately after heterotopic cardiac transplantation, and in the following 5 days, three additional half-dose (250 µg, i.p.) FR70 injections were performed. **(B)** The graft survival data are presented in detail. C3H recipients that underwent transplantation of a heart from B6 mice were either not treated or treated with FR70 (n = 18 mice for not treated group, n = 25 mice for treated group, mean ± SEM, pooled from five independent experiments). The mean survival time (MST) is shown in **Table 1**. A statistical evaluation of the graft survival was performed using Kaplan–Meier curves and compared using log-rank tests, ****p < 0.0001. **(C)** Histologic studies of harvested cardiac grafts were performed with hematoxylin–eosin (HE) staining. Syngeneic B6 heart (Syngeneic), isotype control-treated B6 cardiac allograft (Allo) and FR70-treated B6 cardiac allograft (Allo + FR70) on POD7 are shown (n = 3 mice/group, scale bars: 200 µm).

Animals with grafted cardiac transplants received an isotype control antibody (control) or FR70 (treatment) (**Figure 1A**). As shown in **Figure 1B** and **Table 1**, the IgG-treatment control C3H mice rejected B6 cardiac allografts, with a mean survival time (MST) of 8 days, while the administration of FR70 induced long-term cardiac allograft acceptance, with an MST of about 100 days (p < 0.0001). Grafts from the treatment group recipients showed a completely preserved cardiac structure compared to the control group, with less interstitial infiltration and a similar structure compared to syngeneic recipients with grafts from C3H donors (**Figure 1C**).

**Table 1:** The mean survival time data from the mouse cardiac grafts experiments after treatment with the isotype control or FR70.

Group	*n*	Graft survival days	Mean ± SEM	*p* value to control
Control	18	7 × 2, 8 × 12, 9 × 4	8.1 ± 0.1	N/A
Treatment	25	38, 41, >100 × 23	95.1 ± 3.3	<0.001

A statistical evaluation of the graft survival was performed using Kaplan–Meier curves with a comparison using log-rank tests, and p < 0.05 was considered significant.

### FR70 Administration Reduces the Number of Cytotoxic T Lymphocytes and Down-Regulates the Expression of CTL-Related Genes in GILs and in the Spleen

**Figure 2:**
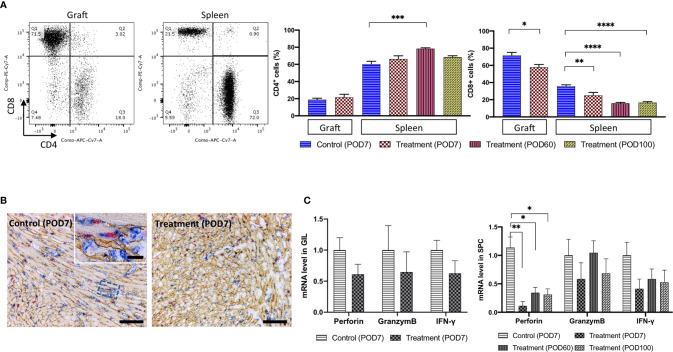
The number of CD8^+^ CTLs and the related mRNA expression were decreased in the GILs and in the SPCs of the FR70-treated recipients. GILs and spleens from isotype control-treated (Allo) and FR70-treated (FR70) groups were collected on POD7 [Control (POD7), Treatment (POD7)]. In addition, spleens from the FR70-treated groups were collected at POD60 and 100 [Treatment (POD60), Treatment (POD100)]. GILs and SPCs were separated and stained with anti-CD4 and CD8 mAb. Samples were subjected to FCM. **(A)** A representative FCM analysis of the CD4 and CD8 in GILs and SPCs is shown (left panel). Gating strategy is shown in **Supplementary Figures 2A**, **B**. Quantification of the CD4 and CD8 in GILs and SPCs by FCM is shown (right panel) (Grafts: n = 4 mice/control group; n = 6 mice/FR70-treated group; Spleens: n = 5 mice/allo group; n = 6 mice/FR70-treated POD7 group; n = 8 mice/FR70-treated POD100 group, mean ± SEM, pooled from three independent experiments). Statistical analysis was determined by Student’s t-test, one-way ANOVA and Tukey’s test. *p < 0.05, **p < 0.01, ***p < 0.001, ****p < 0.0001. **(B)** FR70-treated and control allografts were harvested on POD7 and stained with anti-CD8 (blue), collagen IV (yellowish-brown), and BrdU (red) by triple immunostaining (scale bars: 200 µm). **(C)** Quantitative RT-PCR of mRNA in GILs collected on POD7 from the FR70-treated and control groups (n = 4–5 mice/group) and SPCs collected on POD7 from the control group and on POD7, 60 and 100 from the FR70-treated group (n = 5 mice/group). The mean ± SEM are presented, pooled from three independent experiments with three biological replicates. Statistical analysis was determined by Student’s t-test, one-way ANOVA, and Tukey’s test. *p < 0.05, **p < 0.01.

GILs from the control group and FR70-treated group were analyzed by FCM on POD7 **(** **Figure 2** **)**. GILs from the treatment group had a significantly smaller population of cytotoxic CD8^+^ T cells (but not CD4^+^ T cells) than the control group (**Figure 2A**). In addition, a more detailed immunohistochemical (IHC) analysis of the grafts confirmed that there were fewer focal infiltrations of CD8^+^ lymphocytes in the treatment group than in the control group (**Figure 2B**).

Similarly, SPCs from the syngeneic transplant group, control group and treatment group were analyzed by FCM on POD7. In addition, for the treatment group, we also performed an analysis of the SPCs from POD60 and 100. Animals treated with a control antibody had increased numbers of CD8^+^ cells compared to the syngeneic group (not shown), while the administration of FR70 reduced the number of CD8^+^ cells (**Figure 2A**). The effect was even more pronounced in SPCs in comparison to GILs (30 and 54% decrease in the number of CD8^+^ in SPCs at POD7 and POD60, respectively, *vs* 19% in GILs). In FR70-treated recipients with a long-term survival, the population of CD8^+^ cells in the spleen was stable even at POD100 (**Figure 2A**). Interestingly, the CD4^+^ cell population was significantly decreased on POD7 in the control group, whereas in recipients treated with FR70 the number of CD4^+^ cells increased on POD60 (**Figure 2A**).

Except for the CTL levels checked by FCM in the spleen, the expression of genes related to CTLs, such as perforin, interferon-*γ* and granzyme B, was evaluated by quantitative RT-PCR at POD7, 60 and 100 in SPCs from treatment recipients. The expression of perforin was significantly increased in the spleen in the control group mice, but treatment with FR70 stabilized the expression of this gene to the level observed in the syngeneic model (not shown), with long-lasting efficacy (**Figure 2C**). The expression of interferon-*γ* (IFN-*γ*), a cytokine activating the immune response and produced by, among others, CTLs, was also reduced to some extent after treatment with FR70 (*p* > 0.05), but there was no such effect found for granzyme B expression, possibly due to the increased number of CD4^+^ cells in the spleen. In contrast to the data obtained from the spleen, the expression of the CTL-related genes perforin and granzyme B as well as interferon-*γ* in GILs was not significantly reduced after the treatment with FR70 (**Figure 2C**). This confirms the data obtained from the analysis on CTL levels in GILs and spleen showing most dramatic changes in the spleen and to a lesser extent in GILs.

In previous studies, memory T cells have been reported to be affected by the treatment with FR70 in a cardiac transplant model in presensitized mice. We also performed FCM analysis of effector and central memory cells among CD4^+^ and CD8^+^ in GILs and SPCs after the treatment with FR70, however, none of the memory T cell populations were found to be significantly decreased in the GILs and SPCs after the treatment (**Supplementary Figure 1**). These results may emphasize an important role of activated memory T cells in more aggressive models with presensitization to the graft donor antigens and less impact of these T cell populations in less acute mouse models without presensitization.

### Treatment With FR70 Generates TolDCs

**Figure 3:**
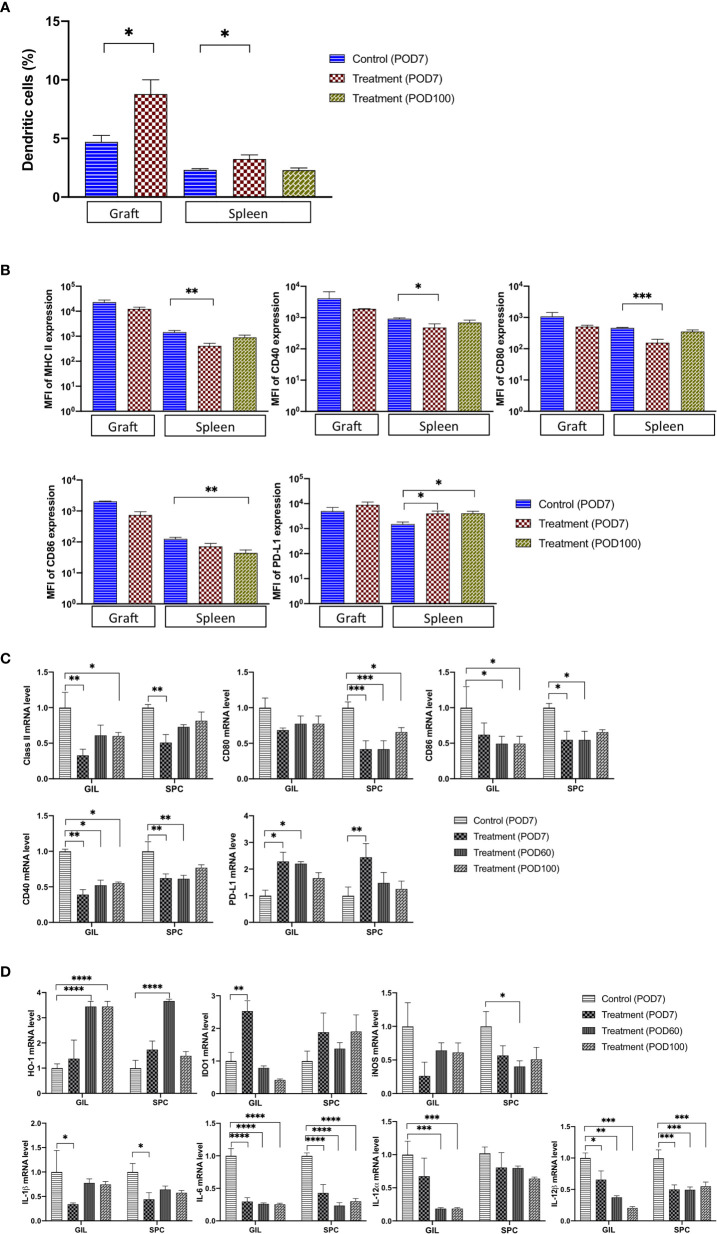
The number of TolDC and the related mRNA expression were significantly changed in graft and spleen of the FR70-treated recipients. **(A)** GILs were collected from control (Allo) and FR70-treated groups on POD7, and spleens were harvested from the control group (Allo) on POD7 and the FR70-treated group on POD7 and 100 for FCM. Gating strategy is shown in **Supplementary Figure 3A** (n = 3–5 mice/group, mean ± SEM, pooled from two independent experiments). Statistical analysis was determined by Student’s t-test, one-way ANOVA, and Tukey’s test. *p < 0.05, **p < 0.01, ***p < 0.001. **(B)** The median fluorescence intensity (MFI) in CD11c and CD11b double-positive cells in graft and spleen was estimated for cells stained with anti-CD40, CD80, CD86, IA/IE, or PD-L1 antibodies and detected by FCM (Grafts: n = 2 mice/control group; n = 3 mice/FR70-treated group; Spleens: n = 4 mice for isotype control group; n = 5 mice/FR70-treated group POD7 and POD100, mean ± SEM, pooled from two independent experiments). Statistical analysis was determined by Student’s t-test, one-way ANOVA, and Tukey’s test. *p < 0.05, **p < 0.01, ***p < 0.001. **(C, D)** Grafts and spleens were collected from the control and FR70-treated groups on POD7. In addition, GILs and SPCs from the FR70-treated groups were collected at POD60 and 100. The mRNA expression was quantified by quantitative RT-PCR (Grafts: n = 4 mice/control group; n = 4 mice/FR70-treated POD7 group; n = 5 mice/FR70-treated POD60 group; n = 5 mice/FR70-treated POD100 group; Spleens: n = 4 mice/isotype control group; n = 5 mice/FR70-treated POD7, 60 and 100 groups, mean ± SEM, pooled from two independent experiments with three biological replicates). Statistical analysis was determined by one-way ANOVA and Tukey’s test. *p < 0.05, **p < 0.01, ***p < 0.001, ****p < 0.0001.

DCs play a pivotal role in the activation of naïve T cells as the most potent antigen-presenting cells (APCs) (10, 15). The interaction between CD70 expressed on APCs and CD27 present on T cells is a critical step in priming effector and memory T cells by DCs (10, 16). In order to explore the effects of FR70 treatment in graft recipients on DCs, we determined the presence of DC cells in grafts and spleen tissue and analyzed the expression of co-stimulatory molecules, such as CD40, CD80, CD86 and major histocompatibility complex (MHC) class II antigens, both in SPCs and GILs. The numbers of CD11c^+^CD11b^+^ double-positive cells representing DCs were significantly increased in graft and spleen during treatment (an increase of 87 *vs* 41% in graft and spleen, respectively; **Figure 3A**). When we evaluated the expression of CD40, CD80, CD86 and MHC class II proteins in CD11c^+^CD11b^+^ cells by FCM, we found that the expression of all of these proteins was lower in the FR70-treated recipients (a statistically significant difference reached in spleen) in comparison to the control group treated with the isotype antibody (**Figure 3B**). In contrast, the PD-L1 expression was significantly higher in CD11c^+^CD11b^+^ DCs in spleen at POD7 (**Figure 3B**), and it also increased slightly in graft compared with the control group. The observed effects appeared to be long-lasting for the PD-L1 and CD86 expression in spleen, since the same significant change in the expression was still present at POD100 (**Figure 3B**). In agreement with these results, we found that the mRNA levels of CD40, CD80, and CD86 and MHC class II antigens were significantly lower in GILs and/or SPCs isolated from the FR70-treated group at POD7 and/or POD60 and 100 than in those isolated from the isotype control group at POD7 (**Figure 3C**). Similar to the results from FCM analysis, mRNA levels of PD-L1 were significantly increased in animals treated with FR70 in GILs and SPCs (**Figure 3C**).

Furthermore, an analysis of the gene expression of regulatory proteins, such as heme oxygenase-1 **(**HO-1) and indoleamine 2,3-dioxygenase (IDO1) (28–32), showed a significant up-regulation of HO-1 expression in GILs and SPCs and IDO1 in GILs from recipients treated with FR70 **(** **Figure 3D** **)**. Interestingly, the effect on the HO-1 expression was much stronger at later stage both in GILs (POD60 and 100) and SPCs (POD60), whereas the expression of IDO1 in GILs was relatively transient, peaking at POD7 and decreasing afterwards. These data strongly suggested that, after treatment with FR70, DCs in graft and spleen retained their tolerogenic immature status.

To confirm that TolDCs developed during FR70 treatment, we tested the effects of FR70 treatment on the gene expression of other TolDC-related proteins, such as iNOS, IL-1β, IL-6, IL-12*α* and IL-12β (16, 33–36). A gene expression analysis using quantitative RT-PCR revealed that all of these genes, in particular IL-6 and IL-12β, were significantly down-regulated in GILs and/or SPCs in mice treated with FR70 compared with the isotype control group (**Figure 3D**), which indicates that blocking CD70 leads to an increase in the number of TolDCs in spleen and grafts.

### Treatment With FR70 Leads to the Generation of Tregs

As reported previously, DCs are able to regulate T cell functions (12), and Tregs are a key controller of immunological tolerance (37). Recruitment of Tregs for the induction of transplant tolerance has been considered an attractive strategy for attenuating the immune response and preventing transplant rejection. We suspected that Tregs were also involved in the CD70 inhibition-mediated prolonged allograft survival. To prove this, we used FCM to determine the number of CD25 and Foxp3 double-positive cells among GILs and SPCs collected from the FR70-treated recipients on POD7, 60 and 100 and the control group on POD7.

**Figure 4:**
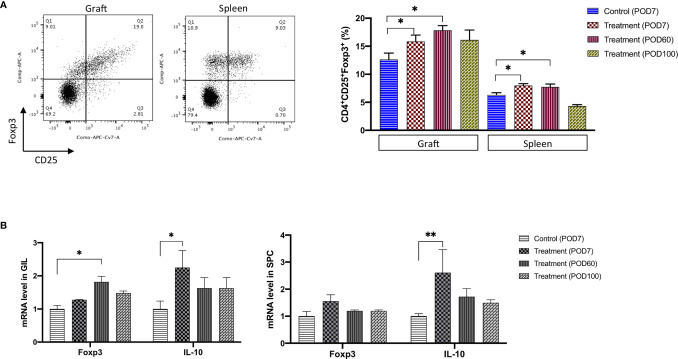
The Treg cell number and the related mRNA expression were significantly increased in graft and spleen of the FR70-treated recipients. **(A)** Grafts and spleens were collected on POD7, 60 and 100. The representative data from Foxp3 and CD25 staining with specific antibodies are presented (left panel). The percentage of CD25^+^ Foxp3^+^ cells among the CD4^+^ GILs and SPCs was determined by FCM (right panel, n = 4–8 mice for each group, mean ± SEM, pooled from two independent experiments). Gating strategy is presented in **Supplementary Figure 3B**. Statistical analysis was determined by one-way ANOVA and Tukey’s test. *p < 0.05. **(B)** Quantitative RT-PCR of the Foxp3 and IL-10 mRNA levels in GILs and SPCs collected at POD7, 60 and 100 (n = 4–5 mice for each group, mean ± SEM, pooled from three independent experiments with three biological replicates). Statistical analysis was determined by one-way ANOVA and Tukey’s test. *p < 0.05, **p < 0.01.

As shown in **Figure 4A**, the population of CD25^+^Foxp3^+^ cells among the CD4^+^ GILs in the FR70-treated group was increased compared with the isotype control recipients (statistically significant increase at POD7 and 60). A similar effect was observed in the spleen cell population (an increase up to 41 and 23% in case of GILs and SPCs at POD60, respectively). The effect gradually declined, and the Tregs count reached the same value as that observed in the control group at POD100 in both graft and spleen tissue (**Figure 4A**). We also evaluated the mRNA expression for Foxp3 using quantitative RT-PCR. The Foxp3 mRNA levels were significantly increased in GILs but less evident in SPCs in the FR70-treated group compared to the isotype control group, and the effect was long-lasting in case of GILs, since increased Foxp3 expression was still detectable at POD100 (**Figure 4B**). These data were consistent with the FCM results, where we also observed a stronger induction of Tregs in GILs.

Tregs produce inhibitory cytokines, such as TGF-*β* and IL-10, and TGF-*β* is involved in the differentiation of Tregs from CD4^+^ cells. We evaluated the TGF-*β* and IL-10 gene expression at the mRNA level in GILs and SPCs to investigate the effects of blocking CD70 on the expression of these cytokines and found that IL-10 expression significantly increased in GILs and SPCs during treatment (POD7), but only a limited and insignificant increase was observed in case of TGF-*β* (**Figure 4B** and not shown). The mRNA expression of IL-10 clearly decreased after treatment was stopped (POD60 and 100), which was in agreement with the observed decrease in the number of Tregs in animals treated with FR70.

Taken together, our results show that treatment with anti-CD70 antibody leads to CD4^+^CD25^+^Foxp3^+^ Tregs’ expansion, in particular in grafts, which may have a critical impact on the immune response after transplantation.

### TolDCs and Tregs Generated by Treatment With FR70 Are Actively Immunosuppressive

**Figure 5:**
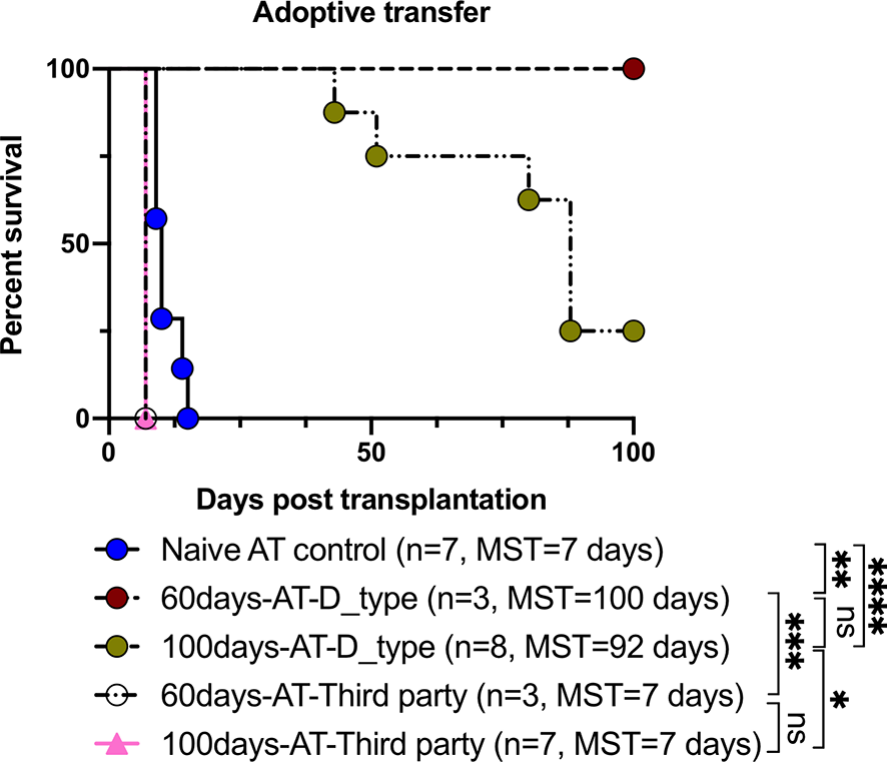
Tolerance induced by FR70 resulted in the acceptance of secondary donor-type cardiac grafts after adoptive splenocyte transfer. The splenocytes harvested at POD60 and 100 from the C3H recipients bearing B6 grafts were adoptively transferred into naïve C3H mice, which were then given B6 (donor type) or BALB/c (third party) cardiac allografts. The graft survival after adoptive transfer of splenocytes is shown (n = 7 mice for Naïve AT control group, n = 3 mice for 60 days-AT-D_type group, n = 8 mice for 100 days-AT-D_type group, n = 3 mice for 60 days-AT-Third party group, n =7 mice for 100 days-AT-Third party group, pooled from two independent experiments). **Table 2** shows the detailed graft survival data. A statistical evaluation of the graft survival was performed using Kaplan–Meier curves with a comparison using log-rank tests. *p < 0.05, **p < 0.01, ***p < 0.001, ****p < 0.0001.

In order to confirm that induced TolDCs and Tregs were directly involved in the mechanism of tolerance, an *in vivo* adoptive transfer study was performed. As shown in **Figure 5** and **Table 2**, the secondary naïve C3H recipients underwent an adoptive transfer of SPCs isolated from mice (the primary recipients treated with FR70) at POD60 and 100, and they showed a survival rate similar to that observed in the treatment group, with MSTs of 100 and 92 days, respectively. In contrast, secondary C3H recipients given an adoptive transfer of SPCs from naïve C3H mice (naïve AT control) rejected the B6 hearts. Furthermore, all naïve C3H recipients with an adoptive transfer of SPCs isolated from FR70-treated primary recipients at POD60 and 100 acutely rejected third-party (BALB/c) mouse cardiac allografts. These data indicate that the administration of FR70 generated regulatory cells in the recipients that were donor-specific.

**Table 2:** The detailed graft survival data after adoptive transfer of splenocytes.

Group	*n*	Graft survival days	Mean ± SEM	*p* value to naïve AT control	*p* value to 60days-AT	*p* value to 100days-AT
Naïve AT control	7	9 × 3, 10 × 2, 14, 15	10.9 ± 1.0	N/A	0.0070	<0.0001
60 days-AT-D_type	3	100 × 3	100 ± 0.0	0.0070	N/A	NS
100 days-AT-D_type	8	43, 51, 80, 88 × 3, 100 × 2	92.8 ± 2.9	<0.0001	NS	N/A
(60 days)-AT-Third party	3	7 × 3	7.0 ± 0.0	0.0027	0.0253	NS
(100 days)-AT-Third party	7	7 × 7	7.0 ± 0.0	0.0003	NS	0.0002

A statistical evaluation of the graft survival was performed using Kaplan-Meier curves with a comparison using log-rank tests, and p < 0.05 was considered significant.

Interestingly, the secondary naïve C3H recipients receiving SPCs isolated at POD60 from the primary recipients treated with FR70 showed no incidents of graft rejection, whereas in those with transplanted SPCs isolated at POD100 we observed a gradual loss of grafts. These results suggested that there were occurring changes in time in the potency of SPCs from FR70 treated animals to mediate immunotolerance in secondary recipients. In order to identify factors important for maintenance of the immunotolerogenic properties of SPCs observed in our model on POD60 and POD100, we categorized all data from our analysis of the immune cells in spleen based on the post-treatment timeline (POD7, POD60 and POD100) (**Table 3**). In animals treated with FR70 there were differences in the number of lymphocytes and DCs, but also qualitative changes in DCs on POD7, 60 and 100. The observed decrease in the number of CTLs did not explain the difference in the tolerogenic properties of SPCs at POD60 and 100, since the decrease was observed to a similar extent at all three PODs. In contrast, a significant increase in the number of Tregs was observed at POD7 and 60, but Treg levels decreased at POD100. Moreover, analysis of DCs showed a significant increase in numbers of DCs only just after the treatment (POD7), but the remaining DCs at POD60 and 100 showed tolerogenic properties with the expression pattern characteristic for TolDCs. More TolDC-related changes in DCs were observed at POD60 than at POD100 (*e.g.* increased HO-1 and decreased iNOS and CD40 expression). Therefore, quantitative and qualitative differences in Tregs and DCs correlate with a stronger tolerogenic potential of SPCs harvested at POD60 than POD100 to support graft survival after secondary transplant. Nevertheless, there were also observed long-lasting significant changes characteristic for TolDCs and tolerogenic microenvironment (decreased CD80/CD86, increased PD-L1 expression and decreased expression of IL-6 and IL-12 cytokines) at POD7, 60 and 100, which explains the ability of SPCs harvested even at POD100 to support a relatively long-term acceptance of allograft in secondary recipients. One would assume that the observed changes in Treg and DC numbers could potentially affect survival of grafts in the primary recipient group, but only very few mice treated with FR70 rejected graft during our study (**Table 1**). Therefore, we conclude that both quantitative and qualitative changes in regulatory cells and microenvironment combined with the reduced number of CTLs were sufficient to maintain a long-term graft tolerance also in the primary recipients (**Supplementary Figure 4**).

**Table 3:** Statistically significant quantitative and qualitative changes in the immune response in spleen of recipients after FR70 treatment on POD7, 60 and 100 in comparison to POD7 of the control recipients.

Compartment	POD7	POD60	POD100
**Spleen**			
**T cell-related**	**↓ CTL number**	**↓ CTL number**	**↓ CTL number**
**CTLs**	↓ perforin mRNA	↓ perforin mRNA	↓ perforin mRNA
	**↑ Treg number**	**↑ Treg number**	
**Tregs**	↑ IL-10 mRNA		
**DC-related**	**↑ DC number**	Not available	
**TolDCs**		↑ HO-1 mRNA	
		↓ iNOS mRNA	
	↓CD40 mRNA/protein	↓ CD40 mRNA	
	↓MHCII mRNA/protein		
	↓ CD80 mRNA/protein	↓ CD80 mRNA	↓ CD80 mRNA
	↓ CD86 mRNA	↓ CD86 mRNA	↓ CD86 protein
	↑ PD-L1 protein	Not available	↑ PD-L1 protein
**Cytokines**	↓ IL-1β mRNA		
	↓ IL-12β mRNA	↓ IL-12β mRNA	↓ IL-12β mRNA
	↓ IL-6 mRNA	↓ IL-6 mRNA	↓ IL-6 mRNA

Cells isolated from spleen on POD60 and 100 were used in a secondary transplant experiment. Long-term tolerogenic effects observed in the primary recipients were caused by time-dependent quantitative and qualitative changes in regulatory cells, which also translated in the tolerogenic memory effect and acceptance of grafts in the secondary transplant recipients; ↓ decreased levels, ↑ increased levels.

## Discussion

The role of CD70 in cardiac transplant acceptance has already been reported (2), but the details of the mechanism underlying this phenomenon still remain to be elucidated. Here we used the mouse model of cardiac grafting without presensitization to dissect the mode of action of anti-CD70 monotherapy in preventing graft rejection (**Supplementary Figure 4**). CTLs and memory T cells play a critical role in cardiac allograft rejection (1, 6, 22, 38). The monotherapy with anti-CD70 antibody induced allograft acceptance by decreasing CTLs to numbers observed in the syngeneic mouse model but did not affect CD4^+^ cells, in agreement with the previously published data (2). Moreover, the therapy did not cause depletion of memory T cells, suggesting that the memory T cells are not involved in the primary response in our model. Interestingly, the models of viral infections in CD70^−/−^ mice showed that the lack of CD70 expression mainly affects effector CD8^+^ but not CD4^+^ nor memory T cells (39), supporting our conclusions that FR70 monotherapy also mainly affected the CTLs.

The observed discrepancy in the response to memory T cells may also be partially due to using mouse strains with a different genetic background or the presensitization increasing the severity of the immune response. For example, the memory T cells involved in cardiac rejection in more acute models with presensitization are able to overcome the benefit of treatment with antibodies (5). The treatment with different antibody combinations, including anti-CD70, showed a dramatic reduction in the number of both CD4^+^ and CD8^+^ memory T cells and prolonged graft survival (4, 5, 39) but were not able to promote the long-term acceptance.

Of note, we used mouse IgG2 FR70 antibody in contrast to the rat FR70 used in other studies, which may also have had an impact on the immune response in the previously described models (5) and might explain some contradictions in the efficacy of FR70 in different studies. The observed effects on CTLs may be due in part to direct targeting of these cells by FR70, which can trigger cytotoxicity and reduce the number of CTLs. Nevertheless, the graft acceptance was durable (lasting >100 days after 1 week of treatment) and cannot be attributed solely to the effector function of FR70. Furthermore, an adoptive transfer of splenocytes harvested 100 days after the treatment with FR70 into the secondary naïve recipient resulted in allograft acceptance. Therefore, it is evident that Tregs and TolDCs from FR70-treated recipients, but not FR70 antibody itself, are responsible for this effect. Consequently, the FR70 works through preventing activation of lymphocytes and keeping the cells in immature tolerogenic state further affecting the immune response, rather than only depleting particular populations of the cells expressing CD70 (**Supplementary Figure 4**).

DCs play a key role in determining the balance between immunity and tolerance, depending on their status. Different strategies for inducing TolDCs in order to promote allograft acceptance or increase immune tolerance are currently being explored (19, 40). We herein report the therapeutic potential of inducing TolDCs *via* CD70 blocking in order to achieve long-term allograft acceptance. The expression analysis of DC markers showed a lower expression of the co-stimulatory molecules and MHC II on DCs in SPCs and GILs from the FR70-treated group recipients in comparison with the control group. In addition, the expression of pro-inflammatory cytokines produced by conventional DCs involved in allograft rejection, in particular IL-6 and IL-12 (38, 41–43), was significantly decreased in our model after treatment with FR70. DCs with a reduced expression of these molecules are defined as DCregs or TolDCs (15, 16). Moreover, DCs from the FR70-treated group expressed higher levels of PD-L1 and HO-1, which also promotes the immature and tolerogenic properties of DCs and mediates induction of Tregs. Consistently, the population of CD4^+^CD25^+^Foxp3^+^ cells increased in the treatment group recipients. The observed effects on the number of regulatory cells were moderate but sufficient to restore the immune balance and induce the long-term acceptance of the cardiac allograft. Interestingly, relatively more TolDCs and Tregs were detected in GILs than SPCs, whereas more dramatic effect on CTLs was observed in SPCs.

Recently, Singh et al. published data on mechanism of a long-term islet allografts tolerance in rhesus macaques (44). Adding apoptotic donor leukocytes infusion to short-lived immunosuppression induced a regulatory network characterized by significant and sustained increase in circulating regulatory myeloid, T and B cells, but also qualitative changes in regulatory cells populations with the most prominent role of Tr1 cells (44). Our results also show that, post-treatment time-dependent quantitative and qualitative changes in Tregs and TolDCs in animals treated with FR70 are responsible for long-term tolerogenic effects in transplant survival and tolerogenic memory effect translating into long-term acceptance of grafts also in secondary transplant recipients. However, we cannot exclude effects of FR70 on other CD70-expressing cell populations, such as monocytes or macrophages, or other types of regulatory cells in cardiac acceptance after FR70 treatment.

In conclusion, the FR70 monotherapy showed moderate but pleiotropic effects on different immune cell populations with the induction of the tolerogenic cells populations and affecting the effector cells, which contributed to restoring the immune response to the level observed in the syngeneic model and, in effect, resulted in preventing the allograft rejection. The detailed molecular mechanism underlying the effects of anti-CD70 therapy needs to be further clarified, but modulation of the immune response with anti-CD70 mAb may be considered for the induction of clinical transplantation tolerance and as a strategy for engendering long-term allograft acceptance.

## Data Availability Statement

The datasets presented in this study can be found in online repositories. The names of the repository/repositories and accession number(s) can be found in the article/**Supplementary Material**.

## Ethics Statement

The animal study was reviewed and approved by Committee on the Ethics of Animal Experiments of the National Research Institute for Child Health and Development, Tokyo, Japan.

## Author Contributions

JZ: Participated in making the research design, performing the research, analyzing the data and writing the article. WQ: Participated in performing the research, analyzing the data. XD: Participated in performing the research, analyzing the data. MF: Participated in making the research design, revising the article for important intellectual content. NI: Participated in making the research design, revising the article for important intellectual content. HU: Participated in making the research design, revising the article for important intellectual content. NT: Participated in making the research design, revising the article for important intellectual content. W-zG: Participated in making the research design, revising the article for important intellectual content. PZ: Participated in making the research design, revising the article for important intellectual content. HH: Participated in making the research design, revising the article for important intellectual content. NN: Participated in making the research design, revising the article for important intellectual content. X-KL: Participated in making the research design, revising the article for important intellectual content. All authors contributed to the article and approved the submitted version.

## Funding

This study was supported by research grants from the Grants of Ministry of Education, Culture, Sports, Science and Technology of Japan (Grants-in-Aid 16K11064, 24/17H04277, 18K08558); and grants from the National Center for Child Health and Development.

## Conflict of Interest

PZ was employed by argenx BV. HH holds ownership interest (including patents) in argenx.

The remaining authors declare that the research was conducted in the absence of any commercial or financial relationships that could be construed as a potential conflict of interest.

## Supplementary Material

Supplementary Figure 1 The population of central and effector memory T cells in the spleen and graft CD4^+^ and CD8^+^ T cells on POD7. **(A)** Grafts were harvested on POD7. The representative data of the CD44 and CD62L staining are presented. The percentage of T_CM_ (CD44^+^CD62L^+^) and T_EM_ (CD44^+^CD62L^−^) cells in the CD4^+^ and CD8^+^ GILs was detected by FCM (n = 4–8 mice for each group, pooled from two independent experiments). Gating strategy is shown in **Supplementary Figure 2A**. The data are shown as the mean ± SEM, and p < 0.05 was considered significant. **(B)** Spleens from syngeneic group and allo group were harvested on POD7, and spleens from FR70 group were collected on POD7 and 100. The representative data of the CD44 and CD62L staining are presented. The percentage of T_CM_ (CD44^+^CD62L^+^) and T_EM_ (CD44^+^CD62L^−^) cells in the CD4^+^ and CD8^+^ SPCs was detected by FCM (n = 4–8 mice for each group, pooled from two independent experiments). Gating strategy is shown in **Supplementary Figure 2B**. The data are shown as the mean ± SEM, and p < 0.05 was considered significant.

Supplementary Figure 2 Gating strategy for the flow cytometric analysis of lymphocytes from graft and spleen. **(A)** Gating strategy for lymphocyte analysis in graft. Doublets were discriminated based on SSC-H and SSC-W, and CD45 positive cells were determined in singlets. Next, living cells were gated on CD45^+^ cells, and then CD3^+^ cells were gated on living cells. Finally, CD3^+^ cells were gated for CD4^+^ and CD8^+^ cells. **(B)** Gating strategy for lymphocyte analysis in spleen. Living cells were gated based on forward (FSC-A) and side scatter (SSC-A). Doublets were discriminated based on SSC-H and SSC-W, and living cells were gated on singlets. Then CD3^+^ lymphocytes were selected. Finally, further analysis of CD4^+^ and CD8^+^ cells on CD3^+^ cells was performed, and the following phenotypic characteristics were used to define different populations of memory T lymphocytes: CD4 or CD8 T_EM_ and T_CM_− CD44^+^CD62L^−^ and CD44^+^CD62L^+^, respectively.

Supplementary Figure 3 Gating strategy for the flow cytometric analysis of DCs (A) and Tregs (B) from graft and spleen. **(A)** Upper panel: Gating strategy for DC analysis in graft. CD45 positive cells were gated for singlets based on SSC-H and SSC-W, and then living cells were gated on singlets and examined for expression of CD11c and CD11b. CD11c^hi^CD11b^hi^ cells representing DCs were used for further analysis. Lower panel: Gating strategy for DC analysis in spleen. Living cells were gated based on forward and side scatter. Doublets were discriminated and living cells were gated on singlets. Living cells were examined for CD11cCD11b double positive cells representing DCs. **(B)** Upper panel: Gating strategy for Treg analysis in graft. Doublets were discriminated based on SSC-H and SSC-W. CD45 positive cells were gated on singlets. Next, living cells were selected, and CD3^+^CD4^+^ lymphocytes were gated on living cells in order to determine CD25^+^Foxp3^+^ double positive Tregs. Lower panel: Gating strategy for Treg analysis in spleen. Doublets were discriminated based on SSC-H and SSC-W, and living cells were gated on singlets. Next, living cells were gated for CD3^+^ lymphocytes, and then CD4 positive cells were gated on CD3^+^ cells. CD4^+^ lymphocytes were analyzed for expression of CD25 and Foxp3.

Supplementary Figure 4 The mode of action of anti-CD70 mAb in mouse cardiac allograft model. Isotype control treated C3H mice acutely rejected B6 cardiac allografts due to involvement of CD8^+^ CTLs, while the treatment with anti-CD70 mAb induced long-term allograft acceptance. Blocking CD70/CD27 signaling by using anti-CD70 mAb caused moderate but pleiotropic effects on different immune cell populations by decreasing CTL numbers and the induction of the tolerogenic cells populations, namely TolDCs and Tregs, which contributed to restoring the immune response to the level observed in the syngeneic mouse model and, in effect, resulted in preventing the allograft rejection.

## Abbreviations

APCs, antigen-presenting cells; CTL, cytotoxic T lymphocyte; DCs, dendritic cells; FCM. flow cytometry; GILs, graft-infiltrating lymphocytes; H&E, hematoxylin and eosin; iPSCs, induced pluripotent stem cells; IFN-*γ*, interferon-*γ*; IHC, immunohistochemistry; MHC, major histocompatibility complex; MST, mean survival time; POD, post-operative day; qRT-PCR, quantitative reverse transcription polymerase chain reaction; SPCs, spleen cells; TNF, tumor necrosis factor; Tregs, regulatory T cells; TolDCs, tolerogenic DCs.
