# Supplementary figures and images for: Monotherapy With Anti-CD70 Antibody Causes Long-Term Mouse Cardiac Allograft Acceptance With Induction of Tolerogenic Dendritic Cells
Source: Front Immunol. 2021 Feb 19;11:555996. doi: 10.3389/fimmu.2020.555996 (PMC7961176; doi:10.3389/fimmu.2020.555996)

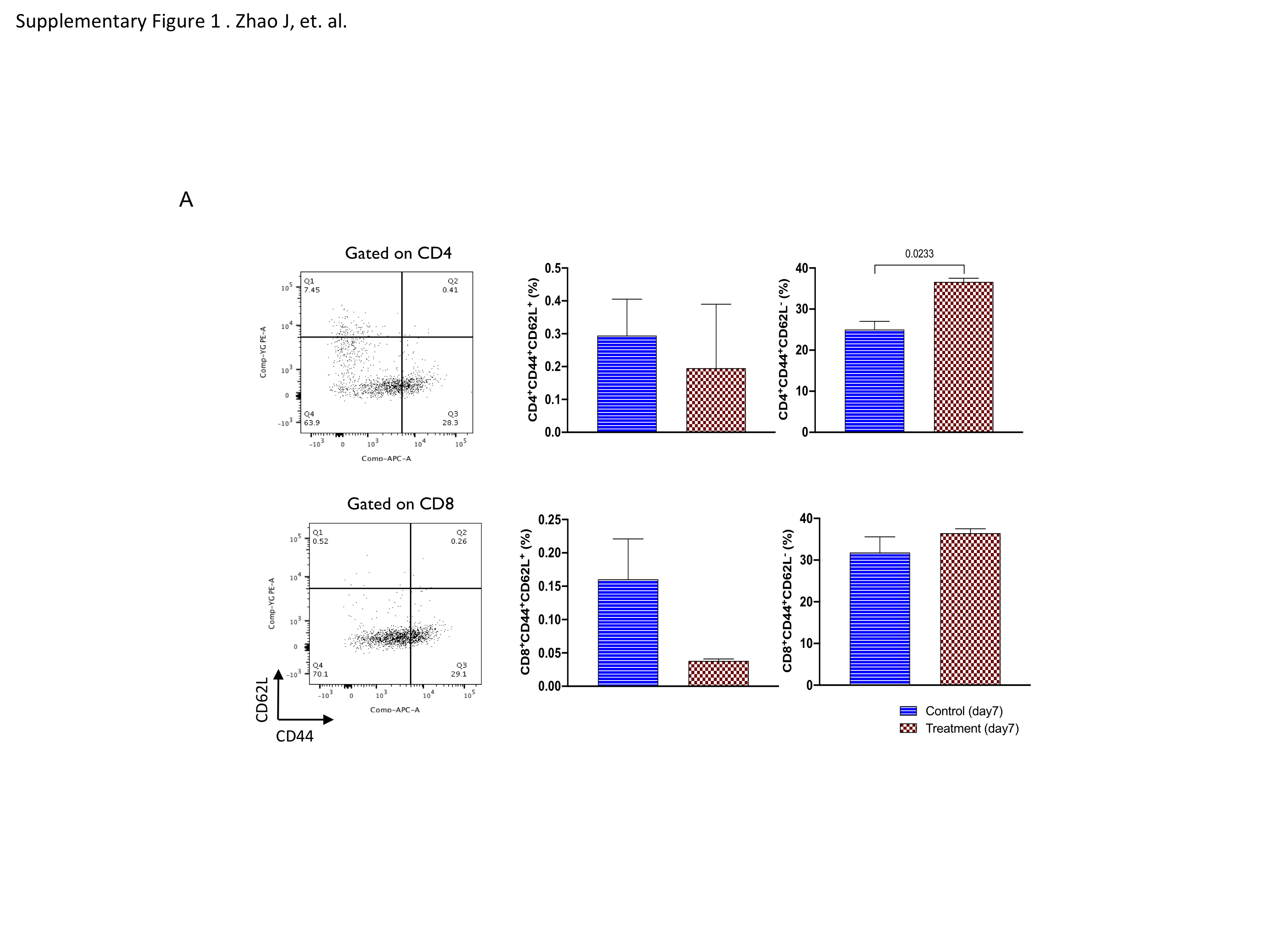

Supplement: Supplementary Figure 1 — The population of central and effector memory T cells in the spleen and graft CD4+ and CD8+ T cells on POD7. (A) Grafts were harvested on POD7. The representative data of the CD44 and CD62L staining are presented. The percentage of TCM (CD44+CD62L+) and TEM (CD44+CD62L−) cells in the CD4+ and CD8+ GILs was detected by FCM (n = 4–8 mice for each group, pooled from two independent experiments). Gating strategy is shown in Supplementary Figure 2A . The data are shown as the mean ± SEM, and p < 0.05 was considered significant. (B) Spleens from syngeneic group and allo group were harvested on POD7, and spleens from FR70 group were collected on POD7 and 100. The representative data of the CD44 and CD62L staining are presented. The percentage of TCM (CD44+CD62L+) and TEM (CD44+CD62L−) cells in the CD4+ and CD8+ SPCs was detected by FCM (n = 4–8 mice for each group, pooled from two independent experiments). Gating strategy is shown in Supplementary Figure 2B . The data are shown as the mean ± SEM, and p < 0.05 was considered significant. [file Image_1.tiff]

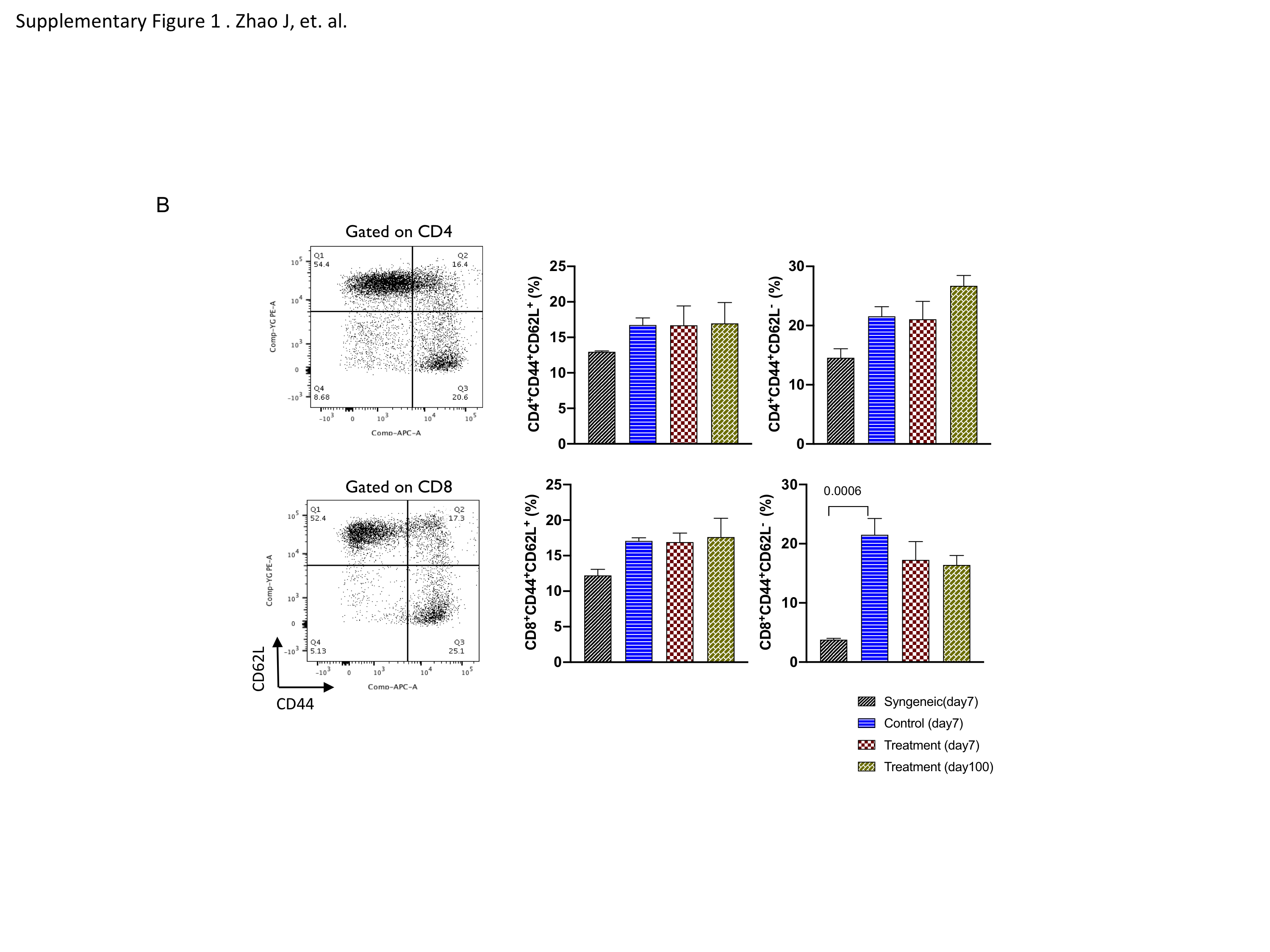

Supplement: Supplementary file 2 [file Image_2.tiff]

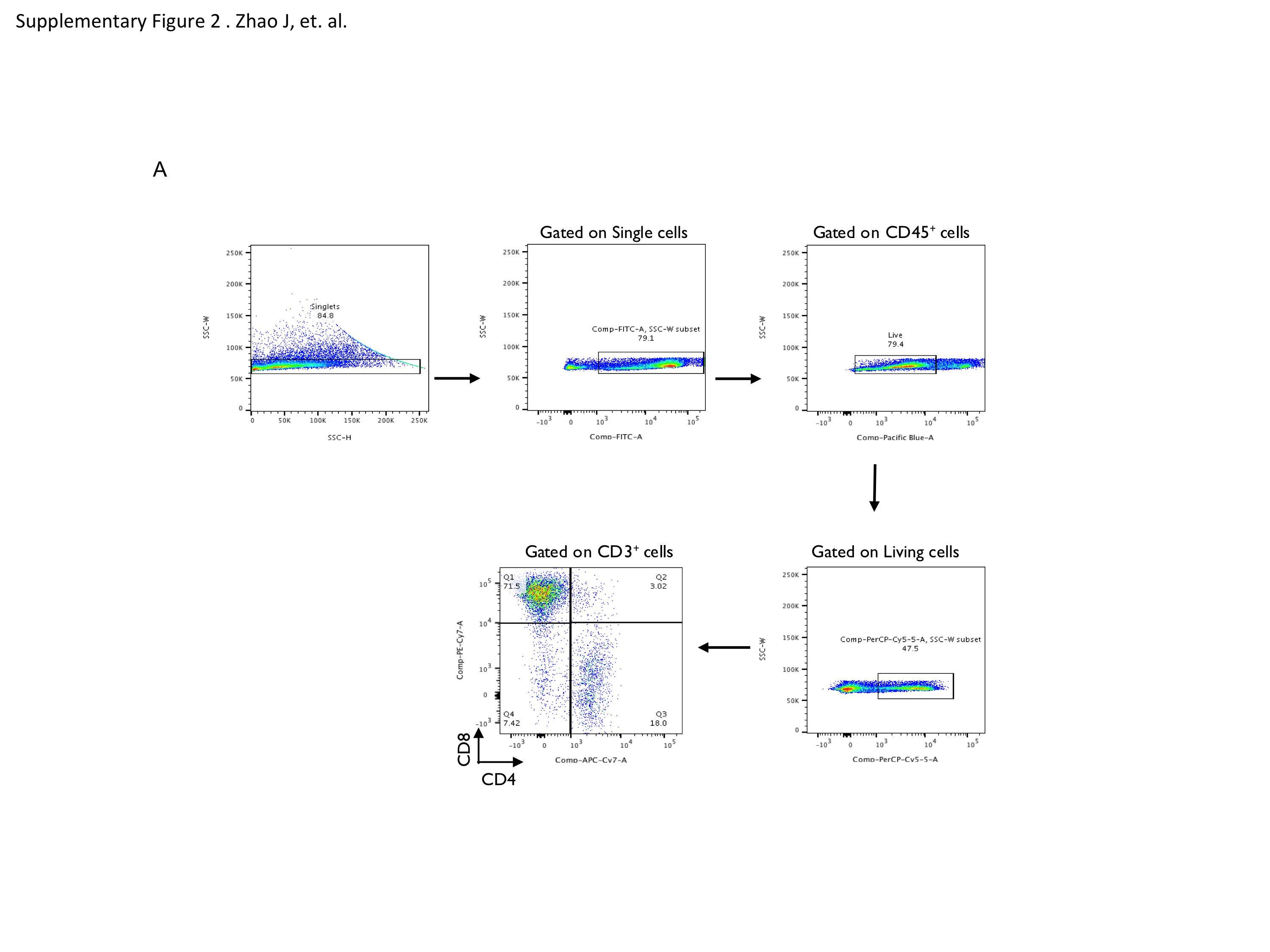

Supplement: Supplementary Figure 2 — Gating strategy for the flow cytometric analysis of lymphocytes from graft and spleen. (A) Gating strategy for lymphocyte analysis in graft. Doublets were discriminated based on SSC-H and SSC-W, and CD45 positive cells were determined in singlets. Next, living cells were gated on CD45+ cells, and then CD3+ cells were gated on living cells. Finally, CD3+ cells were gated for CD4+ and CD8+ cells. (B) Gating strategy for lymphocyte analysis in spleen. Living cells were gated based on forward (FSC-A) and side scatter (SSC-A). Doublets were discriminated based on SSC-H and SSC-W, and living cells were gated on singlets. Then CD3+ lymphocytes were selected. Finally, further analysis of CD4+ and CD8+ cells on CD3+ cells was performed, and the following phenotypic characteristics were used to define different populations of memory T lymphocytes: CD4 or CD8 TEM and TCM− CD44+CD62L− and CD44+CD62L+, respectively. [file Image_3.tiff]

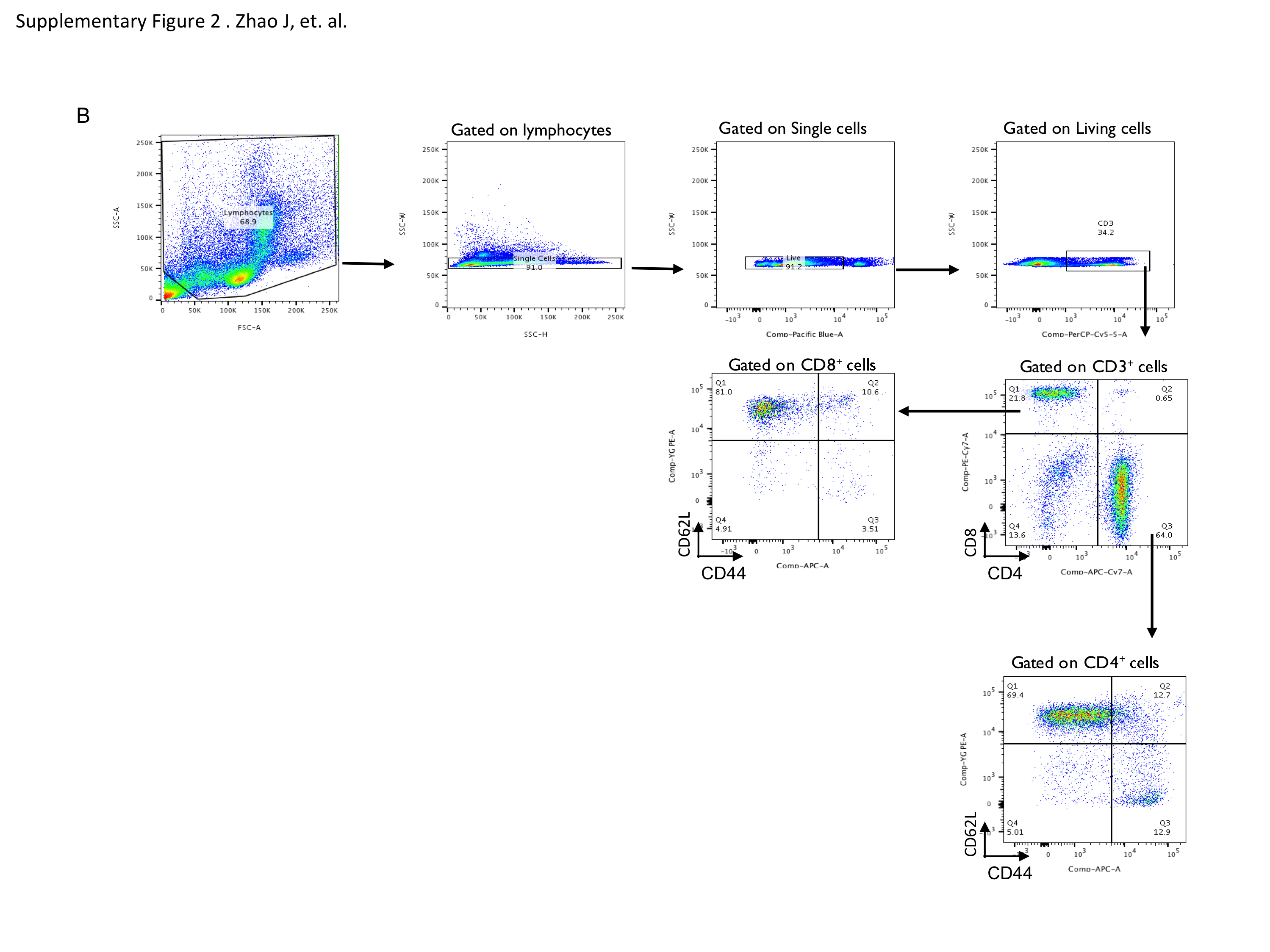

Supplement: Supplementary file 4 [file Image_4.tiff]

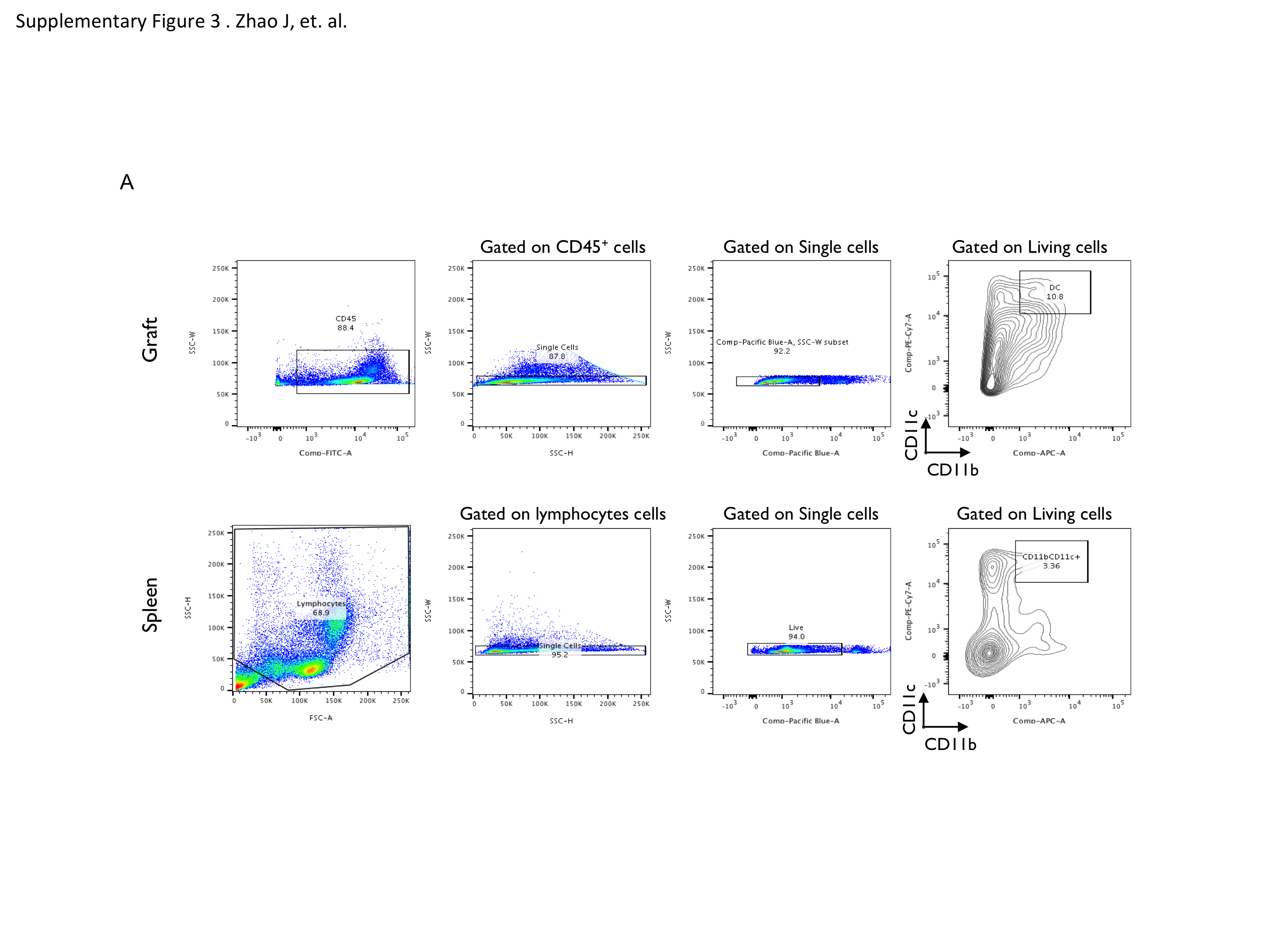

Supplement: Supplementary Figure 3 — Gating strategy for the flow cytometric analysis of DCs (A) and Tregs (B) from graft and spleen. (A) Upper panel: Gating strategy for DC analysis in graft. CD45 positive cells were gated for singlets based on SSC-H and SSC-W, and then living cells were gated on singlets and examined for expression of CD11c and CD11b. CD11chiCD11bhi cells representing DCs were used for further analysis. Lower panel: Gating strategy for DC analysis in spleen. Living cells were gated based on forward and side scatter. Doublets were discriminated and living cells were gated on singlets. Living cells were examined for CD11cCD11b double positive cells representing DCs. (B) Upper panel: Gating strategy for Treg analysis in graft. Doublets were discriminated based on SSC-H and SSC-W. CD45 positive cells were gated on singlets. Next, living cells were selected, and CD3+CD4+ lymphocytes were gated on living cells in order to determine CD25+Foxp3+ double positive Tregs. Lower panel: Gating strategy for Treg analysis in spleen. Doublets were discriminated based on SSC-H and SSC-W, and living cells were gated on singlets. Next, living cells were gated for CD3+ lymphocytes, and then CD4 positive cells were gated on CD3+ cells. CD4+ lymphocytes were analyzed for expression of CD25 and Foxp3. [file Image_5.tiff]

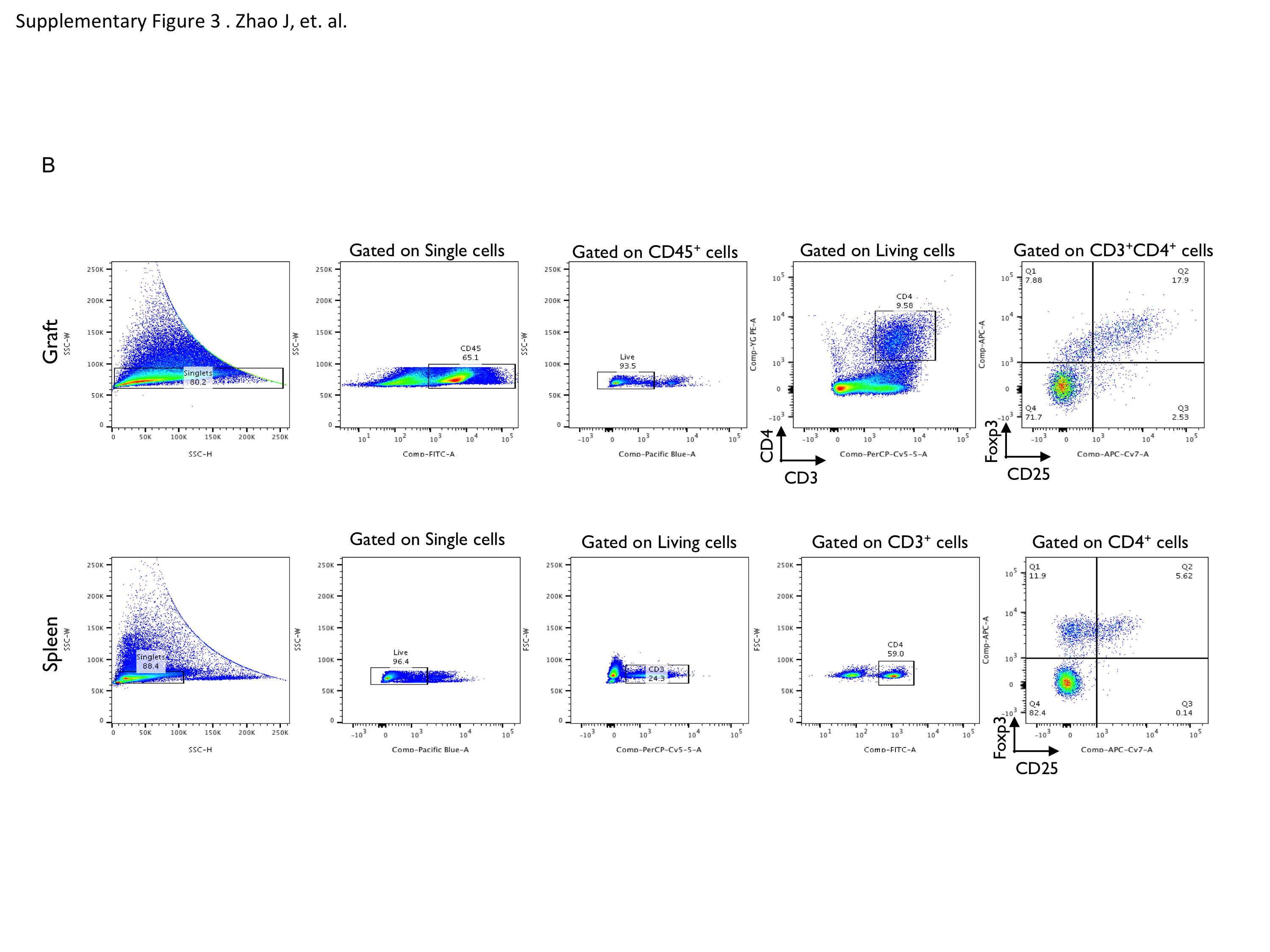

Supplement: Supplementary file 6 [file Image_6.tiff]

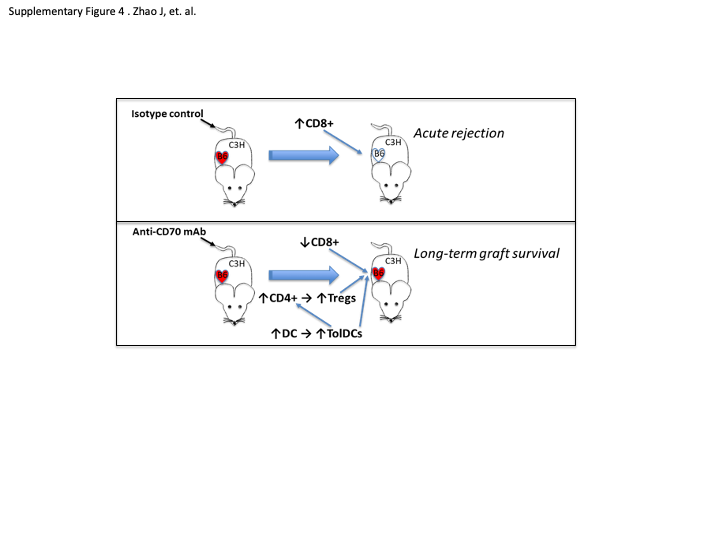

Supplement: Supplementary Figure 4 — The mode of action of anti-CD70 mAb in mouse cardiac allograft model. Isotype control treated C3H mice acutely rejected B6 cardiac allografts due to involvement of CD8+ CTLs, while the treatment with anti-CD70 mAb induced long-term allograft acceptance. Blocking CD70/CD27 signaling by using anti-CD70 mAb caused moderate but pleiotropic effects on different immune cell populations by decreasing CTL numbers and the induction of the tolerogenic cells populations, namely TolDCs and Tregs, which contributed to restoring the immune response to the level observed in the syngeneic mouse model and, in effect, resulted in preventing the allograft rejection. [file Image_7.tiff]
